# Molecular group and correlation guided structural learning for multi-phenotype prediction

**DOI:** 10.1093/bib/bbae585

**Published:** 2024-11-14

**Authors:** Xueping Zhou, Manqi Cai, Molin Yue, Juan C Celedón, Jiebiao Wang, Ying Ding, Wei Chen, Yanming Li

**Affiliations:** Department of Biostatistics, University of Pittsburgh, Pittsburgh, PA 15216, United States; Department of Biostatistics, University of Pittsburgh, Pittsburgh, PA 15216, United States; Department of Biostatistics, University of Pittsburgh, Pittsburgh, PA 15216, United States; Department of Pediatrics, University of Pittsburgh Medical Center Children’s Hospital of Pittsburgh, Pittsburgh, PA 15224, United States; Department of Biostatistics, University of Pittsburgh, Pittsburgh, PA 15216, United States; Department of Biostatistics, University of Pittsburgh, Pittsburgh, PA 15216, United States; Department of Pediatrics, University of Pittsburgh Medical Center Children’s Hospital of Pittsburgh, Pittsburgh, PA 15224, United States; Department of Biostatistics & Data Science, University of Kansas Medical Center, Kansas, KS 66160, United States

**Keywords:** association study, cell-type deconvolution, DNA methylation, feature selection, genomic grouping structure, multi-type prediction

## Abstract

We propose a supervised learning bioinformatics tool, Biological gRoup guIded muLtivariate muLtiple lIneAr regression with peNalizaTion (Brilliant), designed for feature selection and outcome prediction in genomic data with multi-phenotypic responses. Brilliant specifically incorporates genome and/or phenotype grouping structures, as well as phenotype correlation structures, in feature selection, effect estimation, and outcome prediction under a penalized multi-response linear regression model. Extensive simulations demonstrate its superior performance compared to competing methods. We applied Brilliant to two omics studies. In the first study, we identified novel association signals between multivariate gene expressions and high-dimensional DNA methylation profiles, providing biological insights for the baseline CpG-to-gene regulation patterns in a Puerto Rican children asthma cohort. The second study focused on cell-type deconvolution prediction using high-dimensional gene expression profiles. Using Brilliant, we improved the accuracy for cell-type fraction prediction and identified novel cell-type signature genes.

## Introduction

Rapid advances in modern biotechnologies have resulted in the generation of massive omics data, encompassing genetics, transcriptomics, proteomics, epigenetics, and more. These datasets are high-dimensional, where the number of features exceeds the sample size. In association studies, it is common that only a small proportion of features are associated with the outcomes of interest. To identify the truly associated features among high-dimensional candidates, regularization methods are frequently employed to shrink the coefficients of noise to zero.

Another intrinsic characteristic of omics data is the hierarchical genome grouping structures. For example, single nucleotide polymorphisms can be grouped by their harboring genes, and genes can be grouped by functional pathways. Regularized regression methods with high-dimensional predictors that take into consideration of predictor grouping structures have been developed [1, 2]. These methods have been shown to provide superior prediction performance compared to their counterparts that do not consider predictor grouping structures.

Recent research interest has focused on omics studies with multivariate responses, where two distinct approaches are commonly adopted. The first approach involves conducting separate association studies on one response at a time. In contrast, the second approach entails performing a joint association study with all responses together. Statistical methods for the first approach have been extensively studied, with top performers including, but not limited to, the least absolute shrinkage and selection operator (Lasso) [3], the elastic net [4], the group Lasso [2], and the sparse group Lasso [1]. However, a drawback of this approach is that it treats response variables as independent and overlooks the correlation and grouping structures among the responses. Additionally, the first approach typically involves multiple comparisons for post-selection hypothesis testing, which can be stringent and may result in excessive false negatives, especially when the dimension of the responses is high.

The available methods for the second approach are still relatively limited. Li, Nan, and Zhu [5] introduced the multivariate sparse group Lasso (MSGLasso). MSGLasso performs signal selection between high-dimensional multivariate responses and multiple predictors while considering the grouping structures on both levels. It has been shown to outperform univariate response approaches in terms of outcome prediction and feature selection. However, one limitation is that MSGLasso does not explicitly consider the response covariance structure. Wilms and Croux [6] later proposed a multivariate group Lasso with responses’ covariance estimation. This method takes into account the covariance structure of the responses. However, it only considers the group-level sparsity and does not address within-group sparsity.

In this study, we propose an extension of MSGLasso called **Brilliant** (Biological gRoup guIded muLtivariate muLtiple lIneAr regression with peNalizaTion). Similar to MSGLasso, Brilliant leverages the intrinsic genome hierarchy grouping structures in model fitting and predictions. It identifies both important group-level and individual-level association signals. However, Brilliant differentiates itself by explicitly incorporating the covariance structure of multivariate responses. This enhancement allows the proposed Brilliant method to significantly improve the performance of feature selection, outcome prediction, and coefficient estimation compared to MSGLasso and other univariate response approaches.

The rest of the paper is structured as follows: in Section 2, we introduce the detailed algorithm of Brilliant. Section 3 employs simulation studies to demonstrate the performance of Brilliant. Section 4 presents two real data applications, and Section 5 provides a discussion.

## Method

Let $n$ denote the number of samples. Consider the multivariate linear regression model


(1) \begin{align*}& \mathbf{Y = XB + E},\end{align*}

where $\mathbf{Y} = (\mathbf{y}_{1}, \ldots , \mathbf{y}_{q}) \in \mathbb{R}^{n \times q}$ is the response data matrix of $n$ samples and $q$ responses, and $\mathbf{X} = (\mathbf{x}_{1}, \ldots , \mathbf{x}_{p}) \in \mathbb{R}^{n \times p}$ is the predictor matrix of $n$ samples and $p$ predictors. Here, $\mathbf{B} \in \mathbb{R}^{p \times q}$ is the coefficient matrix, and $\mathbf{E}\in \mathbb{R}^{n \times q}$ is the matrix of random errors. We assume that each row vector of $\mathbf{E}$ follows a multivariate normal distribution $N(\mathbf{0}, \mathbf{\Sigma } )$ with a zero mean vector and a $q\times q$ covariance matrix $\mathbf{\Sigma }$.

Suppose $\mathbf{X}$ has $P$ groups and $ \mathbf{Y}$ has $Q$ groups. Together, they introduce $P \times Q$ intersection block groups [5] in the coefficient matrix $\mathbf{B}$. Let $\mathcal{G}$ be the set of all $P \times Q$ groups, and $\mathbf{B}_{g}$ be a single block group in $\mathcal{G}$, $g \in{1,...,G=P\times Q}$. For a group $\mathbf{B}_{g}$, denote its $L_{2}$ norm by $ \left \|\mathbf{B}_{g}\right \|_{2} = \sqrt{ \sum _{\beta _{jk} \in B_{g}} \beta _{jk}^{2}} $.

Brilliant considers the following multi-responses optimization problem.


(2) \begin{align*}& \begin{aligned} & \underset{(\mathbf{B})}{\operatorname{argmin}} \frac{1}{2n} \operatorname{tr}((\mathbf{Y}-\mathbf{X B})^{T}(\mathbf{Y}-\mathbf{X B}) \tilde{\Omega} ) + \\ & \lambda_{1} \sum_{1 \leq j \leq p, 1 \leq k \leq q} |\beta_{j k}|+ \lambda_{2} \sum_{g \in \mathcal{G}} w_{g} \| \mathbf{B}_{g}\|_{2}, \end{aligned}\end{align*}

where $\tilde{\Omega }$ is a working version of the precision matrix $\mathbf{\Omega } = \mathbf{\Sigma }^{-1}$. When $\mathbf{\Omega }$ is a diagonal matrix, Brilliant reduces to MSGLasso. Non-negative tuning parameters $\lambda _{1}$ and $\lambda _{2}$ are for the Lasso penalty and group Lasso penalty, respectively. The non-negative $w_{g}$s are adaptive weights for each individual group. In this paper, we set $w_{g}^{2}$ equal to the total number of entries in the group $B_{g}$.

The working precision matrix $ \tilde{\Omega }$ can be estimated from the data. For instance, when using centralized responses, one can calculate $ \tilde{\Omega }$ by directly invert the sample covariance matrix in a low dimensional setting or by employing graphical lasso [7] in a high-dimensional setting. In this paper, we propose utilizing a block-diagonal working precision matrix. The diagonal blocks are obtained by inverting the sample variance-covariance matrices of responses within each group, while the off-diagonal blocks are set to zero. We show that using a adequately specified $ \tilde{\Omega }$, Brilliant can outperform MSGLasso.

Let $\hat{\mathbf{B}}$ be the solution to (2) and let $\beta _{j k} $ be its entry on the $j$th row and $k$th column. For a given grouping structure $\mathcal{G}$ on $\mathbf{B} $ and a set of tuning parameters, an iterative algorithm, adapted from the mixed coordinate descent algorithm [5] for coefficient estimation is summarized in Algorithm 1. In the original mixed coordinate descent algorithm [5], the outcome covariance structure was not explicitly incorporated into the estimation. Brilliant addresses this limitation by specifically integrating the user-specified working precision matrix into the mixed coordinating algorithm. In Algorithm 1, the estimates of $\beta _{j k} $ by Brilliant depend on $ \tilde{\omega}_{k k} $ and $ S_{j k}$, where $ \tilde{\omega}_{k k} $ is the $kk$-the element ($k$-th diagonal element) of the working precision matrix $ \tilde{\Omega} $ and $ S_{j k} = \mathbf x_{j}^{\top } (\mathbf Y- \mathbf X \mathbf{B}_{(-j k)} ) \tilde{\Omega}_{\cdot k} $ with $ \mathbf{B}_{(-jk)} $ being the $jk$-th element of $\mathbf B$ replaced by zero and $\tilde{\Omega}_{\cdot k}$ being the $k$th column vector of the working precision matrix. Similar to MSGLasso, in each updating iteration, Brilliant updates the whole group to a zero group if $\sqrt{ \sum _{ (j k: \beta _{j k} \in B_{g } )} \left (\left |S_{j k}^{(m-1)}\right | / n-\lambda _{1}\right )^{2}} \leq \lambda _{g }$. Otherwise, for a nonzero updating group, Brilliant update a $\hat \beta _{jk}$ within the group according to three different conditions. (I) If all groups containing $\beta _{jk}$ satisfy $\left \|\hat{\mathbf{B}}_{g-(j k)}^{(m-1)}\right \|_{2}=0$ in the current iteration, where $\hat{\mathbf{B}}_{g-(j k)}^{(m-1)}$ is $\hat{\mathbf{B}}_{g}^{(m-1)}$ with $\hat \beta _{j k}^{(m-1)}$ replaced by 0, then Brilliant updates $\hat \beta _{jk}$ using the Lasso solution; (II) If all groups containing $\beta _{jk}$ satisfy $\left \|\hat{\mathbf{B}}_{g-(j k)}^{(m-1)}\right \|_{2}> 0$ in the current iteration, then Brilliant updates $\hat \beta _{jk}$ using the group Lasso solution; (III) If only some but not all groups containing $\beta _{jk}$ satisfy $\left \|\hat{\mathbf{B}}_{g-(j k)}^{(m-1)}\right \|_{2}=0$ in the current iteration, then Brilliant updates $\hat \beta _{jk}$ using a mixture of Lasso (for groups with $\left \|\hat{\mathbf{B}}_{g-(j k)}^{(m-1)}\right \|_{2}=0$) and group Lasso (for groups with $\left \|\hat{\mathbf{B}}_{g-(j k)}^{(m-1)}\right \|_{2}>0$) solutions. The specific updating equations are detailed in Algorithm 1.

Incorporating the outcome covariance structure can be particularly beneficial when the covariance structure of the outcomes is known a priori or when the outcomes are highly correlated within or between groups. It is important to note that Brilliant incorporates both outcome correlations within groups and across groups, as evidenced by $S_{j k}$.

**Algorithm 1:** Brilliant mixed coordinate descent algorithm

**Input:** Standardize the data
$\sum _{i=1}^{n} y_{ik}= 0$
$\sum _{i=1}^{n} x_{ij}= 0, \sum _{i=1}^{n} x_{ij}^{2}= 1 \mbox{ for } j \in \{1,\dots , j\}, k \in \{1, \dots , q\}$
$ \tilde{\Omega } $ working precision matrix estimated from centralized $ \mathbf{Y}$
**Main Algorithm:**
$\hat \beta _{j k}^{(0)} = 0$ for all ${j \in \{1,\dots , p\}, k \in \{1, \dots , q\}}$
$m \gets 1$ , $\delta ^{(1)} \gets \infty $, $\epsilon \gets $ convergence threshold
**while** $ \delta ^{(m)} {>} \epsilon $ **do**
$j \gets 1$ , $k \gets 1$
**for** j = 1: p **do**
**for** k = 1: q **do**
Calculate $ S_{j k} = \mathbf x_{j}^{\top } (\mathbf Y- \mathbf X \mathbf{B}_{(-j k)} ) \tilde{\Omega}_{\cdot k} $ ⊳ $S_{jk}$ is the partial derivative of the sum of error squares with respect to $\beta _{jk}$
**if** $\sqrt{ \sum _{ (j k: \beta _{j k} \in B_{g } )} \left (\left |S_{j k}^{(m-1)}\right | / n-\lambda _{1}\right )^{2}} \leq \lambda _{g }$ **then** ⊳ Conditions for being updated as zero group(s)
$ \hat{\mathbf{B}}_{g}^{(m)} \gets 0$ with ${\hat \beta _{j k}}^{(m)} \gets 0$ for every $\beta _{j k}^{(m)} \in \mathbf{B}_{g}$
**else if** $\sqrt{ \sum _{ (j k: \beta _{j k} \in B_{g } )} \left (\left |S_{j k}^{(m-1)}\right | / n-\lambda _{1}\right )^{2}}> \lambda _{g }$ **then** ⊳ Conditions for being updated as non-zero group(s)
**if** $\left \|\hat{\mathbf{B}}_{g-(j k)}^{(m-1)}\right \|_{2}=0$ for all groups $g$ containing $\beta _{jk}$**then**
$\hat{\beta }_{j k}^{(m)} \gets \frac{\operatorname{sgn}\left (S_{j k}^{(m-1)}\right )\left (\left |S_{j k}^{(m-1)}\right | -n \lambda _{2}-n \lambda _{1} \right )_{+}}{ \left \| x_{j} \right \|_{2}^{2} \tilde{\omega} _{k k}}$ ⊳ A closed form Lasso solution. Here $\tilde{\omega} _{k k}$ is $kk$-th element of $\tilde{\Omega }$
**else if** $\left \|\hat{\mathbf{B}}_{g-(j k)}^{(m-1)}\right \|_{2}>0$ for all groups $g$ containing $\beta _{jk}$**then**
$\hat{\beta }_{jk}^{(m)} \gets \frac{S_{j k}^{(m-1)}}{\left \|x_{j}\right \|_{2}^{2} \tilde{\omega}_{k k} + n \lambda _{2} / \left \|\hat{\mathbf{B}}_{g}^{(m-1)}\right \|_{2}} $ ⊳ A group Lasso solution
**else if** Some but not all groups $g$ containing $\beta _{jk}$ satisfy $\left \|\hat{\mathbf{B}}_{g-(j k)}^{(m-1)}\right \|_{2}>0$**then**
$\hat{\beta }_{jk}^{(m)} \gets \frac{\operatorname{sgn}\left (S_{j k}^{(m-1)}\right )\left (\left |S_{j k}^{(m-1)}\right | -n \lambda _{2}-n \lambda _{1} \right )_{+}}{\left \|x_{j}\right \|_{2}^{2} \tilde{\omega}_{k k} + n \lambda _{2} / \left \|\hat{\mathbf{B}}_{g}^{(m-1)}\right \|_{2}} $ ⊳ A mixture of Lasso and group Lasso solutions. Notice the numerator is the same as the Lasso solution and the denominator is the same as the group Lasso solution
**end if**
**end if**
**end for** ⊳ for index $k$
**end for** ⊳ for index $j$
$m \gets m + 1$ , $ \delta ^{(m)} = \left \| \hat{\mathbf{B}}^{(m-1)} - \hat{\mathbf{B}}^{(m)} \right \|_{\infty } $ ⊳ Can be replaced by other norm $\left \| \cdot \right \|$
**end while** ⊳ Iterate until $\delta ^{(m)}$ reaches the prespecified precision level

To mitigate false positives, we apply an additional thresholding step to the estimated coefficient matrix $\hat{\mathbf{B}}^{(m)}$ using a non-negative thresholding hyperparameter $b_{thr}$. Specifically, $ \tilde{\beta} ^{(m)}_{jk} = \hat \beta ^{(m)}_{jk} \mathbb{1} (| \hat \beta ^{(m)}_{jk} | < b_{thr} )$, where $1 \le j \le p$ and $1 \le k \le q$. The optimal value of $b_{thr}$ is tuned from the data. More details about selecting $ b_{thr}$ can be found in the Appendix.

## Simulation studies

In our simulation studies, we compared the performance of Brilliant with that of MSGLasso using the R package *MSGLasso* [5] and the univariate method Lasso with the package *glmnet* [8]. The total number of predictors was set to 500, divided into $P=10$ non-overlapping groups, each containing 50 predictors. Predictors within the same group were correlated with either a compound symmetry (CS) or a first-order auto-regression (AR1) structure with a correlation coefficient $\rho = 0.5$. Predictors from different groups were set to be independent. The predictor vector for each subject was independently generated from multivariate normal distributions with the specified correlation structures, marginal variances of 1, and zero means. The total number of responses was set to be 100, separated into $Q=4$ non-overlapping groups, each including 25 responses. For a given grouping structure $\mathcal{G}$, we further assumed that variables within a group were correlated, while variables from different groups were uncorrelated. This is equivalent to stating that the true covariance matrix $\mathbf{\Sigma }$ takes the form of a block-diagonal matrix. The error vector for each subject was also independently generated from multivariate normal distributions with a similar within/between-group correlation structure as the predictor vector and a correlation coefficient $\rho $ taking values in ${0.1, 0.3, 0.5, 0.7, 0.9}$.

The coefficient matrix $\mathbf{B}$ was structured to have a group pattern with $P \times Q = 40$ intersection groups. To introduce group-level sparsity in $\mathbf{B}$, we randomly selected $1/5$ of all groups to be non-zero groups, indicating groups with informative features. To achieve within-group sparsity, we randomly selected $1/2$ of the entries within a non-zero group and set them to 0. The other $1/2$ non-zero values were drawn from a uniform distribution $\operatorname{U} {\left [-3, 1) \cup (1, 3\right ]}$. The matrix $\mathbf{Y}$ was generated based on the multivariate multiple linear regression framework described in (1).

For each of the simulation scenarios, 100 experiments were independently replicated to evaluate the performance. Each experiment contained a training, a validation and a test dataset. We set the training sample size to $200$, and the validation and test sample size to $100$. We applied Brilliant on the training data using a block-diagonal working precision matrix with each diagonal block being the inverse of a within-group sample covariance matrix. A grid-search of selecting the optimal hyperparameters based on the prediction performance on the validation dataset was performed. The final prediction performance was evaluated on the test dataset. The performance of outcome prediction, parameter estimation and feature selection was assessed. Outcome prediction was evaluated by average of the squared differences between the referenced cell fractions and their predicted values, or the mean square error (MSE), defined in (3), where $n$ denotes the sample size in the test set. Feature selection and coefficient estimation were evaluated based on the estimated regression coefficient matrix $\boldsymbol{\widetilde{B}}$. For coefficient estimation, mean absolute estimation error (MAEE) (4) was assessed. For feature selection, we calculated precision, recall and F1 score as formulated in (5)-(7), where $|\mathcal{S}|$ is cardinality of a set $\mathcal{S}$:


(3) \begin{align*} & \textrm{MSE}= \frac{1}{n \times q} \sum_{i=1}^{n} \sum_{k=1}^{q} ( \hat{y}_{ik} - y_{ik} )^{2}. \end{align*}

(4) \begin{align*} & \textrm{MAEE}= \frac{1}{p \times q} \sum_{j=1}^{p} \sum_{k=1}^{q} \left|\tilde{\beta}_{ jk} -\beta_{jk}\right|. \end{align*}

(5) \begin{align*} & \operatorname{Precision} =\frac{\left| \{j k: 1 \leq j \leq p, 1 \leq k \leq q, \tilde{\beta}_{j k} \neq 0 \textrm{ and } \beta_{j k} \neq 0 \} \right|}{\left|\{j k: 1 \leq j \leq p, 1 \leq k \leq q, \tilde{\beta}_{j k} \neq 0 \}\right|}. \end{align*}

(6) \begin{align*} & \operatorname{Recall} =\frac{\left|\{j k: 1 \leq j \leq p, 1 \leq k \leq q, \tilde{\beta}_{j k} \neq 0 \textrm{ and } \beta_{j k} \neq 0 \}\right|}{\left|\{j k: 1 \leq j \leq p, 1 \leq k \leq q, \beta_{j k} \neq 0 \}\right|}. \end{align*}

(7) \begin{align*} & \operatorname{F1} = 2 \times \frac{\operatorname{Precision} \times \operatorname{Recall}}{\operatorname{Precision} + \operatorname{Recall}}.\end{align*}

**Figure 1:**
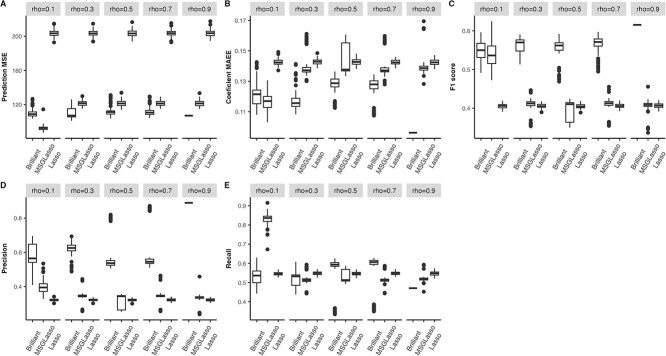
Simulation results of $P = 500$ in $10$ groups, $Q = 100$ in $5$ groups, covariance structure of each predictor group is first-order auto-regressive (AR1) with associated correlation coefficient $\rho =.5$, covariance structure of each error group is AR1 with$\rho =.1$, $0.3$, $.5$, $.7$, or $.9$. Training sample size is 200. (A) MSE metric of prediction error, and lower value means better prediction performance; (B) MAEE of coefficient matrix with lower value suggesting better estimation (C); F1 score for feature selection; (D) Precision for feature selection. (E) Recall for feature selection; for **C**–**E**, higher value indicates better feature selection performance.

Figure 1 summarizes the performance of Brilliant, MSGLasso, and Lasso when the covariance structure is AR1. Brilliant consistently outperforms MSGLasso and the univariate Lasso when the correlation $\rho $ within the same error group is $0.3$ or greater. Among the multivariate approaches, MSGLasso demonstrates the second-best prediction performance (Fig. 1A) after Brilliant. This reaffirms that considering the multivariate response grouping structure can enhance prediction compared to the univariate response approach. It also highlights that incorporating the responses’ covariances can further improve prediction. The corresponding Figure under the CS response covariance structure is given in the Appendix.

Concerning coefficient estimation (Fig. 1B), Brilliant consistently achieves the lowest estimation error, especially when the responses are highly correlated. In terms of feature selection, Brilliant outperforms MSGLasso and Lasso, providing higher precision, recall, and F1 scores when response correlations range from moderate to high (Fig. 1C-E). MSGLasso excels when the correlation $\rho $ is low ($\rho = 0.1$), attributed to the estimation error introduced during the calculation of the precision matrix (inverting the working covariance matrix) in Brilliant.

We also carried out simulation studies to investigate the senarios with overlapping and mispecified grouping structures. The simulation settings and results are detailed in the Appendix. The simulation results showed that Brilliant provided comparable prediction performance to MSGLasso and Lasso under overlapping grouping structures. Notably, the prediction performance of Brilliant tends to improve with a stronger within-group correlation of the error terms and can even surpass MSGLasso and Lasso, even under mis-specified grouping structures. For coefficient estimation and variable selection, Brilliant outperforms MSGLasso and Lasso regardless of the misspecification of grouping structures, especially when the within-group correlation is strong.

## Real data application

We showcase the practical use and performance of Brilliant using real data from two distinct studies: the in-house multi-omics Epigenetic Variation and Childhood Asthma in Puerto Ricans (EVAPR) study [9] and the Framingham Heart Study (FHS) [10, 11]. In the EVAPR study, we collected phenotype, bulk RNA-sequencing (RNA-seq) gene expression, and DNA methylation (DNAm) data from Puerto Rican children aged 9–20 years. Specifically, we focus on the 218 non-asthma controls who have complete data for all predictors and outcomes. For the FHS dataset, sourced from dbGaP (phs000007.v32.p13), it comprises blood microarray expression data for 4100 samples.

### Association analysis of DNAm and gene expression

In the first real data example, Brilliant and its competing methods were applied to bulk RNA-seq gene expression and DNAm data from nasal epithelium tissue samples obtained from 218 non-asthma children subjects. The goal was to identify the baseline association pattern between CpG sites and atopy-related genes.

**Table 1:** Average of pairwise Pearson correlations for genes within each outcome group

Gene Ontology biological process	Number of genes	Correlation
Histone methylation	22	0.690 (0.155)
Energy derivation by oxidation of organic compounds	54	0.618 (0.162)
Transport along microtuble	39	0.860 (0.081)
Positive regulation of cytokine production	37	0.772 (0.102)

* Averages of pairwise Pearson correlations (standard errors).

**Figure 2:**
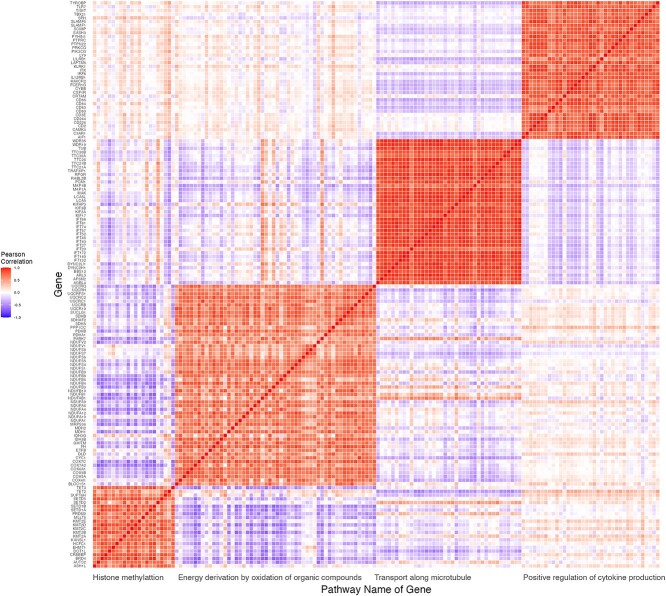
Pearson correlation heatmap of 152 genes. The vertical axis is indexed by gene names. The horizontal axis is indexed by the pathway names that the gene belongs to.

The multivariate responses comprised expressions of 152 genes involved in four atopy-related biological pathways from the Gene Ontology Consortium [12]. Previous studies have implicated cytokines [13] and histones [14] in atopy. To capture these aspects, 37 genes from the ‘positive regulation of cytokine production’ pathway, 22 genes from the ‘histone methylation’ pathway, 54 genes involved in ‘energy derivation by oxidation of organic compounds,’ and 39 genes from the ‘transport along microtubule’ pathway were included. The average pairwise Pearson correlations within pathways was $0.715$ (standard error $0.168$; details in Table 1 and Fig. 2). DNAm information on 1897 CpG sites around the 152 genes (within a region of $1,000$ base pairs up/downstream) was extracted. The top 500 CpGs with the largest variances in their methylation levels ($M$ values) were selected. Log-transformed gene expression transcripts per million (TPM) values and log-transformed methylation $M$ values served as the input responses and predictors for Brilliant and MSGLasso. The grouping of gene responses was based on their pathway memberships, while the grouping of CpG sites was based on the pathways of their harboring or nearest nearby genes. This resulted in four gene groups and four CpG groups, generating 16 (= 4 $\times $ 4) interaction block groups for the underlying regression coefficient matrix. The sizes of CpG groups varied from 91 to 143.

A three-fold nested cross-validation (CV) procedure was implemented for Brilliant and the competing methods. The inner CV was used for selecting the optimal tuning parameters, while the outer CV was employed for generating prediction results and preventing overfitting.

Brilliant outperformed both Lasso and MSGLasso, as evidenced by the smallest prediction error per gene (Table 2). This superiority can be attributed, in part, to Brilliant’s incorporation of additional group structure information for both predictors and outcomes when estimating the regression coefficients. Moreover, the relatively strong correlations between genes within each pathway group played a crucial role in achieving accurate prediction outcomes. Brilliant also outperformed many other supervised learning methods such as Elastic net, group Lasso, and random forest, which fail to incorporate either grouping structure or within-group correlations or both (Table 2).

**Table 2:** Prediction performance of Brilliant and its competing methods in the EVAPR Nasal gene expression and methylation association study

	Brilliant	Lasso	MSGLasso	Elastic	Group	Random
				net	Lasso	forest
SSE						
per	38.91	41.23	41.42	40.31	39.23	93.98
Gene						

* ‘SSE per Gene’ is the total sum of squared errors divided by the number of genes.

**Table 3:** Top association signals between DNAm and gene expression uniquely selected by Brilliant

CpG site	Gene	Gene Chr	CpG position	CpG nearby gene	$\hat{\beta }$	Group	p-value
cg04494844	AUTS2	7	7:70257926	AUTS2	0.0182	i-1	0.0398
cg04494844	NDUFV2	18	7:70257926	AUTS2	0.0138	i-2	0.0069
cg05608790	AUTS2	7	7:69427388	AUTS2	-0.0150	i-1	0.0362
cg05608790	NDUFB9	8	7:69427388	AUTS2	-0.0095	i-2	0.184
cg05608790	PDHB	3	7:69427388	AUTS2	-0.0111	i-2	0.0687
cg20134287	AUTS2	7	7:70061455	AUTS2	-0.0208	i-1	0.0387
cg20134287	NDUFS5	1	7:70061455	AUTS2	-0.0192	i-2	0.0547
cg20134287	PDHA1	X	7:70061455	AUTS2	0.0152	i-2	0.000233
cg19832721	KANSL1	17	17:44249866	KANSL1	0.0114	i-1	0.054
cg19832721	NDUFA4	7	17:44249866	KANSL1	-0.0104	i-2	0.136
cg19832721	NDUFB9	8	17:44249866	KANSL1	-0.0122	i-2	0.113
cg18829443	TET3	2	2:74227916	TET3	-0.0171	i-1	0.0395
cg18829443	NDUFB4	3	2:74227916	TET3	0.0181	i-2	0.00448

* $\hat \beta $ is the estimated regression coefficient. ‘Chr’ stands for chromosome. * CpG positions were mapped to hg19 reference genome. * Group i denotes the genes in the the ‘histone methylation’ pathway. Group 1 denotes the CpG sites within genes in ‘histone methylation’ pathway. Group 2 is the CpG sites within genes in ‘energy derivation by oxidation of organic compounds’ pathway. * The p-values are from fitting multivariate linear regressions for each gene response on all its selected associated CpG predictors.

Brilliant detected 7600 potential associations between CpG methylations and gene expressions, while MSGLasso and Lasso identified 2575 and 1728 associations, respectively. Among the 7600 potential associations selected by Brilliant, 408 were also detected by both Lasso and MSGLasso. Brilliant seems to pick up more association signals compared to MSGLasso and Lasso, likely because it can handle weak signals better. Biological data often contains many weak signals with low signal-to-noise ratios. Incorporating strong correlations between predictors, as observed in previous studies [15], can aid in detecting these weak signals. In this case, Brilliant might spot a weak association signal between a predictor and a response due to the strong correlation between that response and other predictors with strong signals. Such signals tend to be missed MSGLasso and Lasso. The average correlations within the pathway groups further support this idea (within-pathway average absolute correlations range from 0.618 to 0.860). Overall, Brilliant’s ability to utilize response correlations seems to give it an edge in detecting weak association signals. In the Appendix, we demonstrate through a simulation study that Brilliant has a superior ability to detect weak association signals.

In the association block between the CpG group ‘histone methylation’ pathway and the gene group ‘energy derivation by oxidation of organic compounds’ pathway, Brilliant detected 96 association signals between DNAm and gene expression (estimated regression coefficient block given in the Appendix). This block comprised 143 CpG sites in the ‘histone methylation’ pathway and 54 genes in the ‘energy derivation by oxidation of organic compounds’ pathway.

The top association signals uniquely detected by Brilliant are listed in Table 3. Among them, the most of the associated CpG sites are located near genes known as key transcriptional and/or epigenetic regulators, with the associating genes being their targeted regulating genes. For example, the association between *cg05608790* and *NDUFB9* was detected by Brilliant ($\beta _{\textrm{Brilliant}} = -0.0095$). This reflects the regulation of *cg05608790* on its neighboring gene *AUTS2* ($\beta _{\textrm{Brilliant}} = -0.0150$), an epigenetic regulator of a transcriptional network, including *NDUFB9* [16]. Similarly, the association between *cg19832721* and *NDUFA4* ($\beta _{\textrm{Brilliant}} = -0.0104$) reflects the regulatory effects of *cg19832721* on its nearby gene *KANSL1* ($\beta _{\textrm{Brilliant}} = 0.0114$). *KANSL1* is a key gene for producing the histone acetyltransferase 8 (KAT8, also known as MOF) regulatory nonspecific lethal complex, critical for acetylation of nucleosomal histone H4 at lysine 16—a unique histone mark controlling chromatin structure and involved in the regulation of transcription [17].

For this coefficient block, Lasso detected an additional 237 potential CpG–gene associations, while MSGLasso detected only 7 association signals within this block.

### Signature matrix extraction for cell-type deconvolution using bulk gene expression data in blood cell sample

Tissue-level omics studies rely on average effects across a heterogeneous collection of cells. For example, studies on peripheral blood include signals from all underlying cell types. Consequently, tissue-level association studies are prone to confounding due to variations in cell composition across samples [18]. To address this, various cell-type deconvolution algorithms have been proposed to infer cell-type fractions in bulk gene expression data collected from tissue samples [19]. Among these methods, reference-based cellular deconvolution is considered the most accurate and reliable approach [19, 20].

In the second real data application, both the EVAPR and FHS datasets were utilized to predict cell-type proportions for blood neutrophil (Neutro), monocyte (Mono), lymphocyte (Lymph), and eosinophil (Eosino) using bulk gene expression data from blood cell samples. The multivariate responses for both datasets were the fractions of the four cell types for each subject, measured through complete blood cell counts [21]. All four cell-type fractions were combined into one response group for both datasets.

In the EVAPR dataset, predictors were the expression levels of signature genes for each cell type based on the widely used leukocyte gene signature matrix reference (LM22) [22]. These genes were grouped into four predictor groups based on their reference cell-type groups: 64 signature genes in the neutrophil group, 53 in the lymphocyte group, 40 in the monocyte group, and 39 genes in the eosinophil group. The TPM counts of predictors underwent log transformation before applying the Brilliant algorithm.

The FHS dataset predictors included blood microarray gene expressions from 4100 samples, and these genes were grouped into four groups based on the leukocyte signature genes available in the dataset (Details in Appendix). The microarray gene expression data were also log-transformed.

Brilliant’s prediction performance was evaluated within each dataset and across the two datasets. For internal prediction within each dataset, a three-fold nested cross-validation was performed on each dataset to select the optimal hyperparameters and avoid overfitting. For cross-dataset prediction, FHS data were used as the training data, and EVAPR data were used as the test data. A three-fold cross-validation was performed on the FHS data to select the optimal hyperparameters. The prediction results are evaluated by the Pearson correlations between the predicted and referenced cell fractions (scatter plots given in Appendix). The correlations range from $0.692$ to $0.896$.

In the comparison with other commonly used reference-based cell-type deconvolution methods, as conducted in the study by Cai *et al*. [23], including CIBERSORT/CIBERSORTx [22], DeconRNASeq [24], DCQ [25], DeCompress [26], DSA [27], dtangle [20], Ensemble Deconvolution [23], Gene Expression Deconvolution Interactive Tool [28], Estimating the Proportions of Immune and Cancer cells [29], Fast And Robust DEconvolution of Expression Profiles [30], hybrid-scale proportions estimation [31], Immune Cell Deconvolution in Tumor tissues [32], and SCDC methods including non-negative least squares and inverse sum of squares [33], all results were based on the LM22 reference. The FHS dataset served as the training data, and the EVARP dataset was used as the test data.

The comparison results, shown in Fig. 4, reveal Spearman’s rank correlation, a conventional performance evaluation metric in the cell-type deconvolution field, between the predicted and referenced cell fractions for each of the aforementioned methods. Although not originally designed as a cell-type deconvolution algorithm, Brilliant achieved the highest average Spearman’s correlation (the vertical bars in Fig. 4) across the four cell types. The MSE between the predicted and referenced cell fractions for each method was also calculated (see Appendix), with Brilliant yielding the smallest average MSE across cell types among all methods. It is worth noting that Brilliant differs from many conventional deconvolution methods in that it necessitates knowing the true cell proportions for training. This requirement sets it apart because other deconvolution methods don’t utilize this information, making direct comparisons somewhat challenging. Here, we’re simply introducing Brilliant as a signature matrix extraction tool and demonstrating its ability to accurately select predictive gene signatures specific to cell types when benchmark reference data with known cell proportions are accessible. The newly identified signature genes can then be leveraged to improve the prediction performance of other deconvolution methods. Additional analyses of the EVAPR and FHS data using MSGLasso and Lasso, such as the Spearman’s correlations, and their comparison to the Brilliant’s results are given in the Appendix. The Spearman correlations are similar between Brilliant, MSGLasso, and Lasso. This indicates that a significant part of the prediction accuracy of Brilliant, MSGLasso, and Lasso can be attributed to the extra known knowledge about cell proportions in the benchmark data.

**Figure 3:**
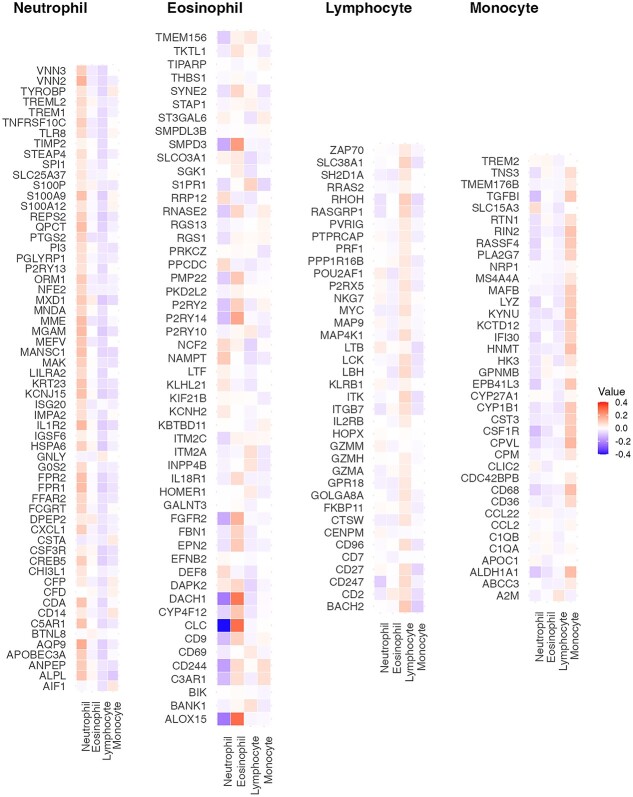
Heatmap of coefficient matrix estimated by Brilliant using the FHS data. The matrix has been divided into four blocks based on the signature genes of each cell type. The four blocks contain the signature genes of Neutrophil, Eosinophil, Lymphocyte, and Monocyte cells, respectively.

**Figure 4:**
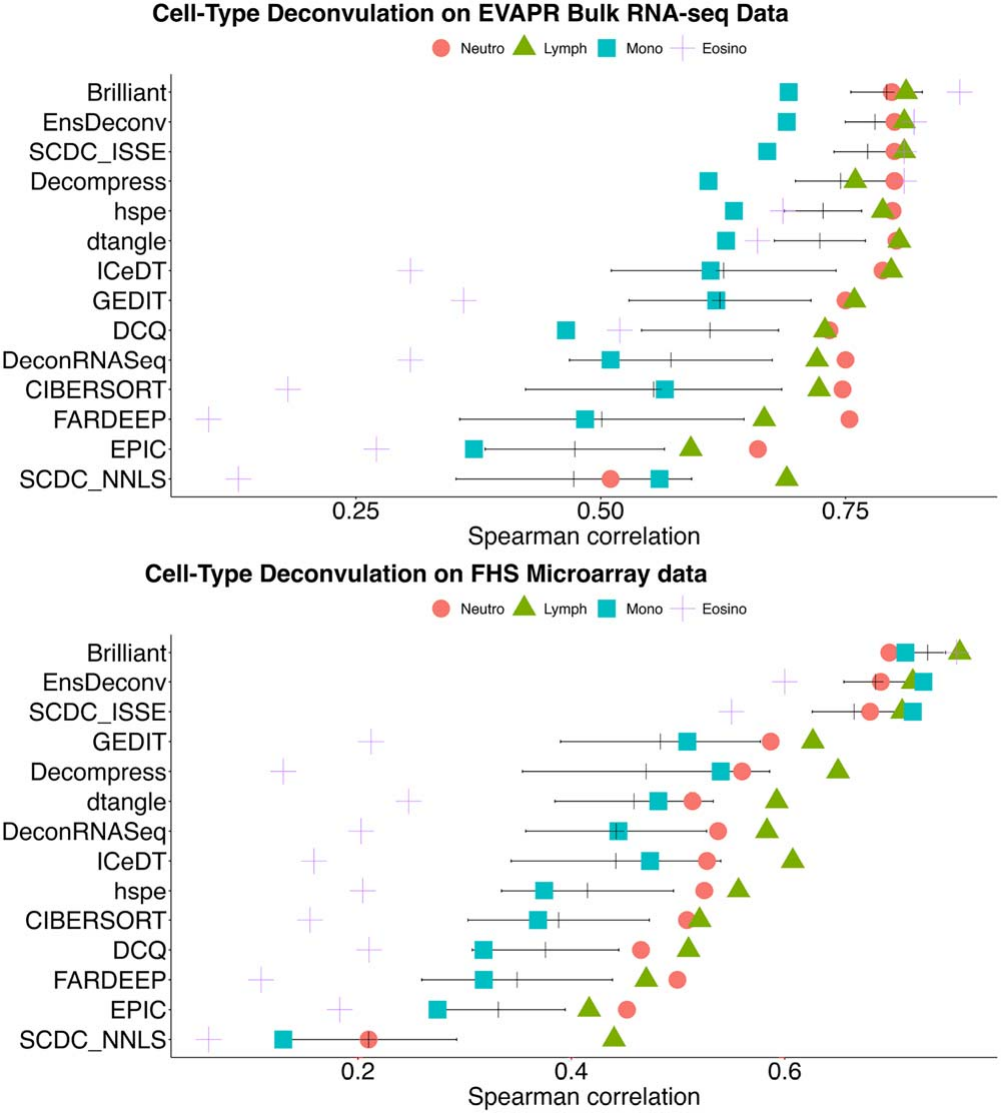
Comparison of cell-type fraction prediction using Brilliant and different deconvolution methods. Neutro: neutrophil; Mono: monocyte; Lymph: lymphocyte; Eosino: eosinophil. The black vertical bar on each row shows the mean of Spearman’s correlations, and the horizontal line presents the range of one standard error below/above the mean.

The feature selection performance of Brilliant was evaluated by comparing the estimated coefficient matrix in the final model (built on FHS data) with the LM22 reference gene matrix. The heatmap in Fig. 3 displays the coefficient matrix estimated by Brilliant, divided into four sub-matrices based on the signature genes of each cell type. As anticipated, for each cell type, the signature genes in the LM22 reference have the highest estimate magnitudes in their corresponding cell-type categories (indicated by darker red color in the corresponding column). Among the 188 genes selected in the final model, Brilliant correctly identified cell types for 144 signature genes. In other words, the highest proportioned cell type in the LM22 matrix is also estimated by Brilliant to have the highest coefficient magnitude across four cell types for these 144 genes. For another 20 genes, the cell type with the second-largest estimated coefficient magnitude is the highest proportioned cell type in the LM22 reference. These results demonstrate that Brilliant is capable of identifying the most informative features associated with the outcomes of interest.

## Conclusion and discussion

In this paper, we introduced an analytical tool called Brilliant for variable selection and outcome prediction in the context of multivariate response and high-dimensional predictor data. Brilliant leverages the intrinsic grouping structures of both predictors and responses, conducting group-level and within-group variable selection. Additionally, it explicitly incorporates the covariance structure of the multivariate responses. Compared to existing methods, Brilliant demonstrates significant improvements in coefficient estimation accuracy, feature selection, and outcome prediction performance. Notably, Brilliant’s approach eliminates the need for iteratively estimating the precision matrix, in contrast to methods such as that proposed by [34]. Instead, it requires a one-step estimation of a working covariance structure based on the user-specified grouping structure for the responses.

We applied Brilliant to two case studies: a methylation-to-gene expression association study and a study predicting cell-type fractions using gene expression. Brilliant’s adaptability is evident, making it easily extendable to address a variety of high-dimensional multivariate-response and multiple-predictor problems. Potential applications include, but are not limited to, single-cell multiomics, expression quantitative trait loci (eQTL) mapping, multivariate phenome-wide association studies (PheWAS), neuroimaging-genomic association studies, and numerous other areas. We anticipate exploring Brilliant’s wide-ranging applications in future research endeavours.

It is important to note that while biologically meaningful grouping structures can often enhance prediction performance, there are instances where this might not hold true, especially when there are signal cancellations within groups. For instance, pathways can sometimes contain inhibitory structures where genes within the pathway are negatively correlated. This negative correlation can nullify the overall effect of the pathway as a cohesive entity. Therefore, it is advisable for end-users to conduct further investigation, particularly on groups containing many negatively correlated features, to better understand their impact on prediction outcomes.

An R package implementing Brilliant is available to download at https://github.com/XuepingZhou/Brilliant.

**Key Points**

- We introduce Brilliant, a bioinformatics tool designed for feature selection and outcome prediction with high-dimensional genomic predictors and multi-phenotypic responses.
- Brilliant specifically incorporates genome and/or phenotype grouping structures, as well as phenotype correlation structures, in feature selection, effect estimation, and outcome prediction.
- We applied Brilliant to a gene expression and DNA methylation association study and a cell-type deconvolution prediction using high-dimensional gene expression profiles.
- Brilliant has a wide range of applications to biomedical studies involving multivariate outcomes, including eQTL, PheWAS, and neuroimaging-genomic association studies.
- An R package is implemented to help researchers in similar studies with multi-phenotype predictions.

## Supplementary Material

Brilliant_Appendix_accepted_10162024_bbae585
