# Supplementary material for: Molecular group and correlation guided structural learning for multi-phenotype prediction
Source: Brief Bioinform. 2024 Nov 14;25(6):bbae585. doi: 10.1093/bib/bbae585 (PMC11562839; doi:10.1093/bib/bbae585)
Supplement: Brilliant_Appendix_accepted_10162024_bbae585 [file brilliant_appendix_accepted_10162024_bbae585.pdf]

Journal Title Here, 2022, pp. i–viii

doi: DOI HERE
Advance Access Publication Date: Day Month Year
Paper

PAPER

Appendix for "Molecular Group and Correlation Guided Structural Learning for Multi-Phenotype Prediction"

Xueping Zhou,[1] Manqi Cai,[1] Molin Yue,[1] Juan C. Celedón,[2] Ying Ding,[1] Wei Chen[2,*] and Yanming Li[3,*]

[1]Department of Biostatistics, University of Pittsburgh, Pittsburgh, 15216, PA, USA, [2]Department of Pediatrics, University of Pittsburgh Medical Center Children's Hospital of Pittsburgh, Pittsburgh, 15224, PA, USA and [3]Department of Biostatistics & Data Science, University of Kansas Medical Center, Kansas, 66160, KS, USA

[*]Corresponding author. Wei Chen, Department of Pediatrics, University of Pittsburgh Medical Center Children's Hospital of Pittsburgh, Pittsburgh, PA 15224, USA, E-mail:wei.chen@chp.edu; Yanming Li, Department of Biostatistics & Data Science, University of Kansas Medical Center, 3901 Rainbow Boulevard, Kansas City, KS 66160, USA, E-mail:yli8@kumc.edu

FOR PUBLISHER ONLY Received on Date Month Year; revised on Date Month Year; accepted on Date Month Year

Abstract

This Appendix provides proofs of technical results, additional tables and figures for the simulation and real data analyses.

Appendix A: Proofs of technical results

Brilliant objective function

Assume $\mathbf{Y}|\mathbf{X} \sim N(\mathbf{XB}, \mathbf{\Sigma})$, the log-likelihood of data can be written as follows.

$$\begin{aligned}&\ln L(\mathbf{B} \mid \mathbf{Y}, \mathbf{X}, \mathbf{\Sigma})\\ &= -\frac{1}{2}\{C - n\ln(|\mathbf{\Omega}|) + \mathrm{tr}[(\mathbf{Y}-\mathbf{XB})^\top(\mathbf{Y}-\mathbf{XB})\mathbf{\Omega}]\}\end{aligned} \tag{1}$$

Given the working precision matrix $\tilde{\mathbf{\Omega}}$, the penalized log likelihood can be written as below.

$$\begin{aligned}\widehat{\mathbf{B}} = \underset{(\mathbf{B})}{\mathrm{argmin}}\frac{1}{2n}\,\mathrm{tr}\left((\mathbf{Y}-\mathbf{XB})^\top(\mathbf{Y}-\mathbf{XB})\tilde{\mathbf{\Omega}}\right) +\\ \lambda_1\sum_{1\le j\le p, 1\le k\le q}|\beta_{jk}| + \lambda_2\sum_{g\in\mathcal{G}}\|\mathbf{B}_g\|_2.\end{aligned} \tag{2}$$

Derivative of Brilliant objective function

The objective function consists of three terms. We take the derivative of each term with respect to β_{jk} separately. Then, we will combine the gradient or sub-gradient together.

Take derivative of the first term with respect to β_{jk}

$$\frac{\partial Q(\mathbf{B})}{\partial\beta_{jk}} = -2\mathbf{x}_j^\top(\mathbf{Y}-\mathbf{XB})\tilde{\mathbf{\Omega}}_{\cdot k}. \tag{3}$$

Let $\mathbf{B} = \mathbf{B}_{(-jk)} + \mathbf{B}_{(jk)}$, where $\mathbf{B}_{(-jk)}$ being the jk-th entry of $\mathbf{B}$ replaced by zero and $\mathbf{B}_{(jk)}$ being all but the jk-th entry of $\mathbf{B}$ replaced by zeros. Define S_{jk} as shown below in equation (4).

$$S_{jk} = \mathbf{x}_j^\top(\mathbf{Y}-\mathbf{XB}_{(-jk)})\tilde{\mathbf{\Omega}}_{\cdot k}. \tag{4}$$

We can re-write equation (3) as

$$\frac{\partial Q(\mathbf{B})}{\partial\beta_{jk}} = -2\times\left(\mathbf{S}_{jk} - ||\mathbf{x}_j||_2^2\beta_{jk}\tilde{\omega}_{kk}\right). \tag{5}$$

Take derivative of the second and third term with respect to β_{jk}

For a coordinate β_{jk}, when $L(\mathbf{B})$ is differentiable at β_{jk},

$$\begin{aligned}\frac{\partial\ln L(\mathbf{B})}{\partial\beta_{jk}} &= -S_{jk}/n + \|\mathbf{x}_j\|_2^2\beta_{jk}\tilde{\omega}_{kk}/n +\\ &\lambda_1\,\mathrm{sgn}(\beta_{jk}) + \lambda_2\beta_{jk}/\|\mathbf{B}_g\|_2.\end{aligned} \tag{6}$$

For general case, the derivative of the second term or $|\cdot|$ penalty can be written as follows.

$$\lambda_1\,\mathrm{sgn}(\beta_{jk}) = \begin{cases}-\lambda_1 & \text{if } \beta_{jk} < 0,\\ [-\lambda_1, \lambda_1] & \text{if } \beta_{jk} = 0,\\ \lambda_1 & \text{if } \beta_{jk} > 0.\end{cases}$$

For general case, the derivative of the third term in the objective function or the derivative of $\|\cdot\|_2$ penalty are shown below.

$$\begin{aligned}&\lambda_2\beta_{jk}/\|\mathbf{B}_g\|_2\\ &= \begin{cases}\lambda_2\beta_{jk}/\|\mathbf{B}_g\|_2 & \text{if } \forall\beta_{jk}\ne 0, \text{where } \beta_{jk}\in\mathbf{B}_g,\\ [-\lambda_2, \lambda_2] & \text{if } \forall\beta_{jk} = 0, \text{where } \beta_{jk}\in\mathbf{B}_g.\end{cases}\end{aligned}$$

© The Author 2022. Published by Oxford University Press. All rights reserved. For permissions, please e-mail: journals.permissions@oup.com

Combine the derivative of the three terms

$$\frac{\partial \ln L(\mathbf{B})}{\partial \beta_{jk}} = \begin{cases} -S_{jk}/n + \|\mathbf{x}_j\|_2^2 \beta_{jk}\tilde{\omega}_{kk}/n - \lambda_1 + \lambda_2\beta_{jk}/\left\|\mathbf{B}_g\right\|_2, \\ \quad \text{if } \beta_{jk} < 0, \\ \text{subgradient, if } \beta_{jk} = 0, \\ -S_{jk}/n + \|\mathbf{x}_j\|_2^2 \beta_{jk}\tilde{\omega}_{kk}/n + \lambda_1 + \lambda_2\beta_{jk}/\left\|\mathbf{B}_g\right\|_2, \\ \quad \text{if } \beta_{jk} > 0. \end{cases}$$

Solution to the Brilliant objective function

Set derivative to 0 and solve for β_{jk} with the corresponding constraint, we can get

$$\hat{\beta}_{jk} = \begin{cases} \frac{S_{jk}/n+\lambda_1}{\|\mathbf{x}_j\|_2^2\tilde{\omega}_{kk}/n+\lambda_2/\left\|\mathbf{B}_g\right\|_2} & \text{if } S_{jk} < -n\lambda_1, \\ 0 & \text{otherwise}, \\ \frac{S_{jk}/n-\lambda_1}{\|\mathbf{x}_j\|_2^2\tilde{\omega}_{kk}/n+\lambda_2/\left\|\mathbf{B}_g\right\|_2} & \text{if } S_{jk} > n\lambda_1. \end{cases}$$

Or, in one unified form:

$$\hat{\beta}_{jk} = \frac{sgn(S_{jk})(|S_{jk}| - n\lambda_1)_+}{\|\mathbf{x}_j\|_2^2\tilde{\omega}_{kk} + n * \lambda_2/\left\|\mathbf{B}_g\right\|_2}. \tag{7}$$

For those who wish to delve deeper into the topic, we have included a comprehensive explanation below.

Details when $\beta_{ij} < 0$

$$-S_{jk}/n + \|\mathbf{x}_j\|_2^2\beta_{jk}\tilde{\omega}_{kk}/n - \lambda_1 + \lambda_2\beta_{jk}/\left\|\mathbf{B}_g\right\|_2 = 0$$

$$\beta_{jk} = \frac{S_{jk}/n + \lambda_1}{\|\mathbf{x}_j\|_2^2\tilde{\omega}_{kk}/n + \lambda_2/\left\|\mathbf{B}_g\right\|_2}.$$

With the constraint $\beta_{jk} < 0$, or $\frac{S_{jk}/n+\lambda_1}{\|\mathbf{x}_j\|_2^2\tilde{\omega}_{kk}/n+\lambda_2/\left\|\mathbf{B}_g\right\|_2} < 0$, we have

$$S_{jk} < -n\lambda_1. \tag{8}$$

Details when $\beta_{ij} > 0$

$$-S_{jk}/n + \|\mathbf{x}_j\|_2^2\beta_{jk}\tilde{\omega}_{kk}/n + \lambda_1 + \lambda_2\beta_{jk}/\left\|\mathbf{B}_g\right\|_2 = 0$$

$$\beta_{jk} = \frac{S_{jk}/n - \lambda_1}{\|\mathbf{x}_j\|_2^2\tilde{\omega}_{kk}/n + \lambda_2/\left\|\mathbf{B}_g\right\|_2}.$$

With the constraint $\beta_{jk} > 0$, or $\frac{S_{jk}/n-\lambda_1}{\|\mathbf{x}_j\|_2^2\tilde{\omega}_{kk}/n+\lambda_2/\left\|\mathbf{B}_g\right\|_2} > 0$, we can get

$$S_{jk} > n\lambda_1. \tag{9}$$

Details on $\beta_{jk} = 0$

We will see different scenarios involving $\beta_{ij} = 0$.
S1. The group containing β_{jk} is not zero groups. This suggests that the Lasso (L_1) $|\cdot|$ penalty is not differentiable, but the $\|\cdot\|$ penalty is differentiable for all groups containing β_{jk}. Set the derivative to 0, we get

$$S_{jk}/n = \lambda_2\beta_{jk}/\left\|\mathbf{B}_g\right\|_2 + \|\mathbf{x}_j\|_2^2\beta_{jk}\tilde{\omega}_{kk}/n + n\lambda_1 u, \tag{10}$$

where $|u| < 1$. When $u \geq 0$, $S_{jk} \geq n\lambda_1$. When $u < 0$, $S_{jk} < -n\lambda_1$. So the solution is the same as that in the above session for $\beta_{jk} \neq 0$) or Equation (7).
S2. The group containing β_{jk} is a zero group. Let $\mathcal{G}^*_{jk} = \{g : \beta_{jk} \in \mathbf{B}_g \in \mathcal{G}, \|\mathbf{B}_g\|_2 > 0\}$. Let $\mathbf{B}_{g0}$ be the zero group containing β_{jk}. Let $|u| < 1$, and $\|\mathbf{v}\|_2 < 1$. Set the derivative to 0, we have

$$-S_{jk}/n + \|\mathbf{x}_j\|_2^2\beta_{jk}\tilde{\omega}_{kk}/n + \lambda_1 u + \lambda_2\nu_{jk} + \lambda_2\beta_{jk}/\left\|\mathbf{B}_g\right\|_2 = 0$$

$$\beta_{jk} = \frac{S_{jk}/n - u\lambda_1 - \nu_{jk}\lambda_2}{\|\mathbf{x}_j\|_2^2\tilde{\omega}_{kk}/n + \lambda_2/\left\|\mathbf{B}_g\right\|_2} = 0$$

$$S_{jk}/n = u\lambda_1 + \nu_{jk}\lambda_2.$$

For each $(jk : \beta_{jk} \in B_{gi})$,

$$\begin{aligned} S_{jk}/n - \mu_{jk}\lambda_1 &= \nu_{jk}\lambda_2 \\ (S_{jk}/n - \mu_{jk}\lambda_1)^2 &= {\nu_{jk}}^2{\lambda_2}^2. \end{aligned} \tag{11}$$

We can see $\hat{\mathbf{B}}_{g0} = 0$ if

$$\sum_{(jk:\beta_{jk}\in B_{gi})} (S_{jk}/n - \mu_{jk}\lambda_1)^2 = \sum_{(jk:\beta_{jk}\in B_{gi})} \nu_{jk}^2\lambda_2^2$$

$$\sum_{(jk:\beta_{jk}\in B_{gi})} (S_{jk}/n - \mu_{jk}\lambda_1)^2 = \lambda_2^2.$$

Discuss on a case by case basis,

$$\begin{cases} \sum_{(jk:\beta_{jk}\in B_{gi})}(S_{jk}/n - \lambda_1)^2 \leq {\lambda_2}^2 & 1 > u \geq 0, \\ \sum_{(jk:\beta_{jk}\in B_{gi})}(S_{jk}/n + \lambda_1)^2 > {\lambda_2}^2 & -1 < u < 0. \end{cases}$$

We can get that $\hat{\mathbf{B}}_{g0} = 0$ if

$$\sqrt{\sum_{(jk:\beta_{jk}\in B_{g0})} (|S_{jk}|/n - \lambda_1)_+^2} \leq \lambda_{g0}. \tag{12}$$

Appendix B: More details about the simulation study

B.1 Details of the simulation study under non-overlapping groups condition

For $p = 500$ and $q = 100$, the simulation results under the first order autoregressive (AR1) covariance structure are presented in the main article. The results under the compound symmetry (CS) covariance structure are given in Figure 5. We observe similar performance. Specifically, Brilliant achieves improved prediction and coefficient estimation compared to MSGLasso and univariate Lasso. Brilliant also shows significant improved feature selection performance, especially in F1 score and precision.

B.2 Simulation study under overlapping groups condition

To evaluate the performance of our algorithm in scenarios where different predictor and response groups overlap, we conducted the following simulation study. In this simulation, we set $p = 35$ and $q = 35$. Both predictors and responses were set to be within two overlapping groups: variables 1-25 were in one group, while variables 11-35 formed the second group. Predictors within each group were simulated from a multivariate normal distribution with zero means, zero marginal variances, and an AR(1) covariance structure and a correlation coefficient $\rho = 0.5$. The errors $\mathbf{E}$ were independently generated in the same way except that we allow the correlation coefficient ρ vary from 0.1 to 0.9. The non-zero beta coefficients were simulated from a uniform

Table 1. Group Structures used by Brilliant and MSGLasso in the overlapping group simulation setups.

	**Predictor Group**	**Outcome Group**
**Brilliant 1**	X1-X35	Y1-Y35
**Brilliant 2**	X1-X25, X26-X35	Y1-Y25, Y26-Y35
**Brilliant 3**	X1-X25, X26-X35	Y1-Y10, Y11-Y35
**Brilliant 4**	X1-X10, X11 -X25, X26-X35	Y1-Y10, Y11 -Y25, Y26-Y35
**MSGLasso***	X1-X25, X11-X35	Y1-Y25, Y11-Y35

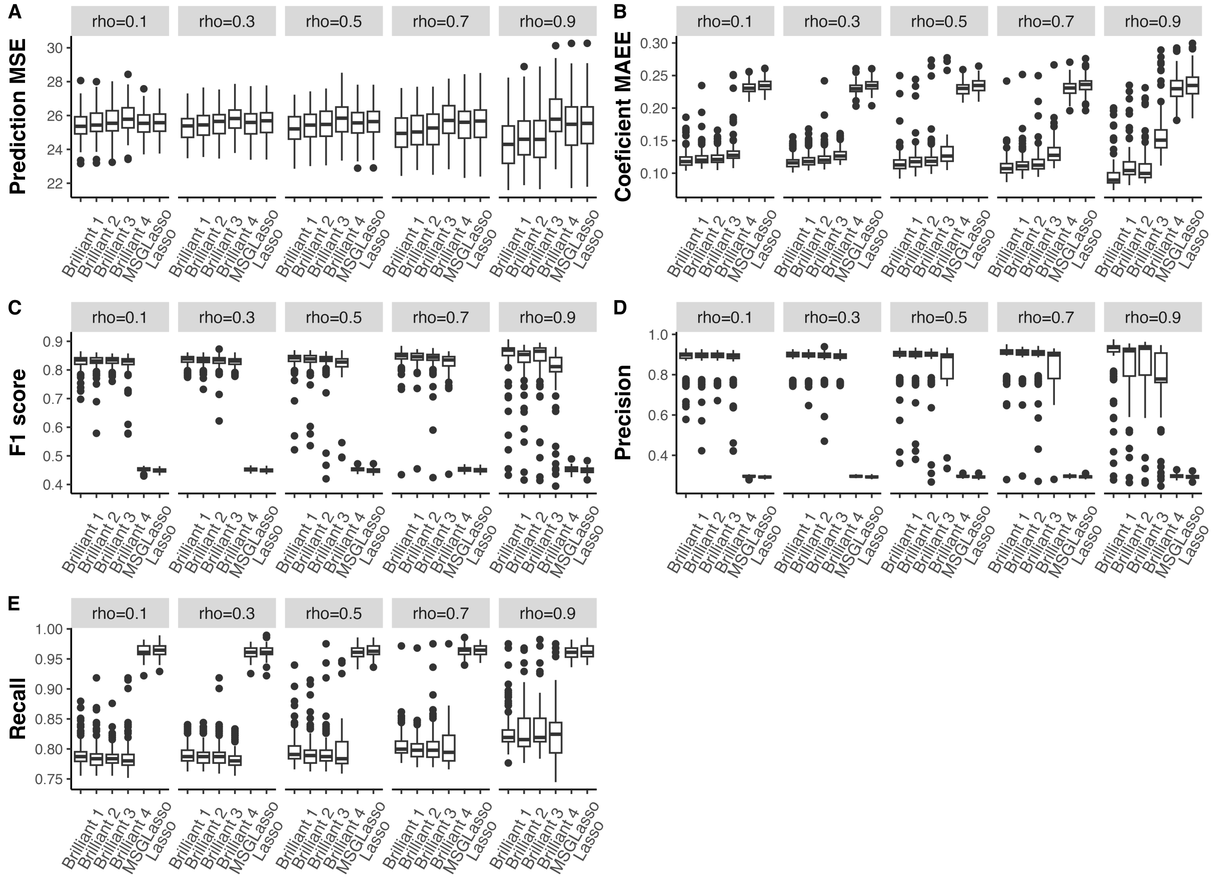

Fig. 1. Simulation results for overlapping group structures. **A**. Mean squared prediction error (MSE), where lower values indicate better prediction performance. **B**. Mean absolute estimation error of the coefficient matrix (MAEE), with lower values suggesting better estimation. **C**. F1 score for feature selection. **D**. Precision for feature selection. **E**. Recall for feature selection. For **C-E**, higher values indicate better feature selection performance.

distribution on $[-5, 0) \cup (0, 5]$. The multivariate responses were then generated from $\mathbf{Y} = \mathbf{XB} + \mathbf{E}$.

For each setting, we generated a training dataset with 200 samples, a validation dataset with 100 samples, and a test dataset with a 100 samples. We fit the Brilliant (with both correctly specified and mis-specified grouping structures), MSGLasso (with the correctly specified grouping structure), and Lasso using the training dataset. The validation dataset were used for selecting the optimal tuning parameters. The prediction was carried on the test dataset. The setting was replicated with 100 independent experiments. We evaluated the performance of variable selection, coefficient estimation, and outcome prediction based on the outputs from these 100 experiments. We considered four mis-specified grouping structures for Brilliant, as shown in Table 1 below. The simulation results are shown in Figure 1 below.

Prediction performance is shown in Figure 1.A. Brilliant gave comparable prediction performance to MSGLasso and Lasso under overlapping grouping structures. It is notable that the prediction performance of Brilliant tends to improve with a stronger within-group correlation of the error terms, and can be better than the MSGLasso and Lasso even under mis-specified grouping structures. For coefficient estimation (Figure 1.B) and variable selection (Figure 1.C-E), Brilliant outperforms MSGLasso and Lasso regardless the misspecification of grouping structures, especially when within-group correlation is strong.

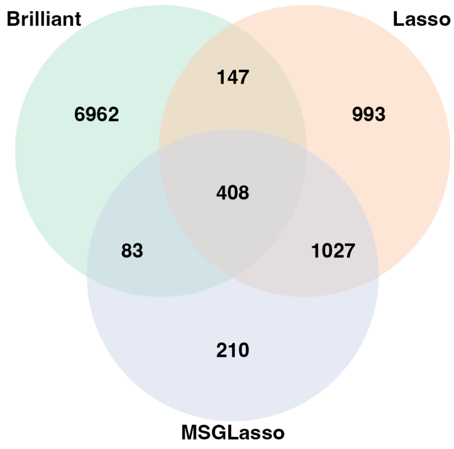

Fig. 2. Venn diagram of numbers of association signals detected by Brilliant and the competing methods in the EVAPR study.

fig:application1.scatter

Appendix C: Further explanation of Lasso feature selection performance

We use a simply simulation study to demonstrate that Lasso is susceptible to selecting more FP signals when there exist correlated outcomes. In this example, we simulated a group of 100 predictors and 40 outcomes. The predictors were generated using the multivariate normal distribution with covariance matrix following compound symmetry (cs) structure with $\rho = 0.2$. The outcomes were simulated from the multivariate normal distribution with covariance matrix following compound symmetry structure and $rho = 0.9$. Then we replaced $Y1$ with $Y1 + X1 + X2$. There were 25 observations in the training dataset. In this case, although both Lasso and Brilliant select the true signals, namely $Y1 - X1$ and $Y2 - X2$. Lasso identify an additional 331 false positives (FPs), while Brilliant only have 6 more FPs (Figure 6).

It is noticeable that Brilliant has lower recalls than Lasso and MSGLasso in the specified working group structures. This is because by dividing the two overlapping groups into more smaller intersection blocks, Brilliant actually increases the risk of selecting false negatives by potentially missing one or more intersection blocks. However, Brilliant demonstrates a significantly lower rate of false positives, thus yielding higher precision compared to MSGLasso and Lasso. MSGLasso is known to produce numerous false positives outside the union of the overlapping groups, while Lasso is also prone to generating many false positives within the groups. Additionally, Brilliant gave smaller parameter estimation error and comparable prediction performance to MSGLasso and Lasso in this overlapping group setting.

Appendix D: More figures for the EVAPR study

The numbers of association signals detected by Brilliant and the competing methods in the EVAPR study are provided in Figure 2

To demonstrate Brilliant's superior ability to detect weak association signals, we conducted a simulation study with correlated weak signals in the coefficient matrix. The simulation setup consisted of 200 predictors divided into 4 groups, each containing 50 variables, and 100 outcome variables divided into 4 groups, each containing 20 variables. The correlation among variables within each predictor group followed a compound symmetry (CS) structure with a correlation of 0.6, while the correlation within each outcome group followed a CS structure with a correlation of 0.75.

In the simulation, we assumed that 37.5% (7500) of the coefficients in the regression coefficient matrix were non-zero, while the remaining 62.5% (12500) were zero. The non-zero coefficients were drawn from uniform distributions Unif[-0.5, 0) and Unif(0, 0.5). Additionally, in each column of the coefficient matrix, the coefficient with the largest absolute value was multiplied by a factor of 3. This resulted in 100 strong signals with a signal-to-noise ratio of approximately 1.25 and 7400 correlated weak signals with a signal-to-noise ratio of approximately 0.25.

The dataset consisted of 200 training samples, as well as 100 validation and test samples each. This simulation setup was repeated 100 times, and cross-validation was used to select the optimal parameters for each method.

The simulation results, as shown in Figure 7, indicate that Brilliant selected significantly fewer false negatives compared to MSGLasso and Lasso (2000 fewer false negatives than MSGLasso and 3500 fewer false negatives than Lasso). Moreover, Brilliant exhibited comparable rates of false positives (a few hundred more than MSGLasso and Lasso). Overall, Brilliant selected thousands more signals than MSGLasso and Lasso, with a majority of these signals being true weak signals. These findings support our speculation about Brilliant's capability of effectively identifying correlated weak signals that are often missed by MSGLasso and Lasso. This is particularly relevant in real omics data studies.

Heatmap of the estimated regression coefficient block between the "histone methylation" pathway and the "energy derivation by oxidation of organic compounds" pathway is depicted in Figure 3.

Appendix E: More details for the FHS study

In the LM22 reference matrix, all entries are positives with higher value indicating higher expression level of the gene in the corresponding cell type. For each gene, we set it as the signature gene for the cell type with the largest value in the reference matrix. The number of available signature genes in the EVAPR and FHS dataset are summarized in Table 2. Table 3 summarizes the top performers of cell type deconvolution for the EVAPR dataset.

Figure 8 gives the scatter plots of predicted v.s. referenced cell type fractions and their Pearson correlations.

Figure 4 compares the cell-type fraction prediction mean squared error (MSE) between Brilliant and different deconvolution methods.

Appendix F: Additional simulation studies

Appendix G: Implementation of Brilliant

In our simulation and real data studies, we applied a cross-validation procedure on the percentiles of the absolute values of the estimated coefficients, to select the optimal hard-threshold value for b_thr. To elaborate, once we obtained the estimated coefficient matrix, we further hard-threshold all its entries by a grid of percentiles (e.g., 10%, 20%, ..., 90%, 100%) of their absolute values, i.e. $\tilde{\beta}_{jk}^{(m)} = \hat{\beta}_{jk}^{(m)} \mathbb{1}(|\hat{\beta}_{jk}^{(m)}| < b_{thr})$. The optimal

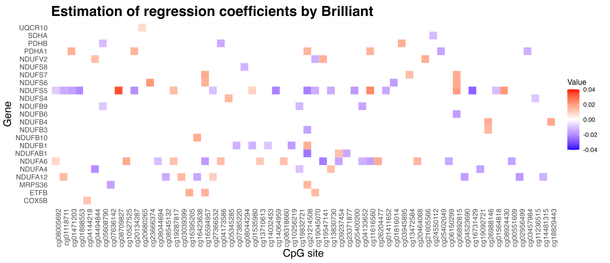

Fig. 3. Brilliant estimation of regression coefficient block corresponds to the associations between the 143 Cpg sites near genes from the “histone methylation” pathway and the 54 genes from the “energy derivation by oxidation of organic compounds” pathway. Only CpG sites and genes with at least one non-zero coefficient estimate are visualized in the graph.

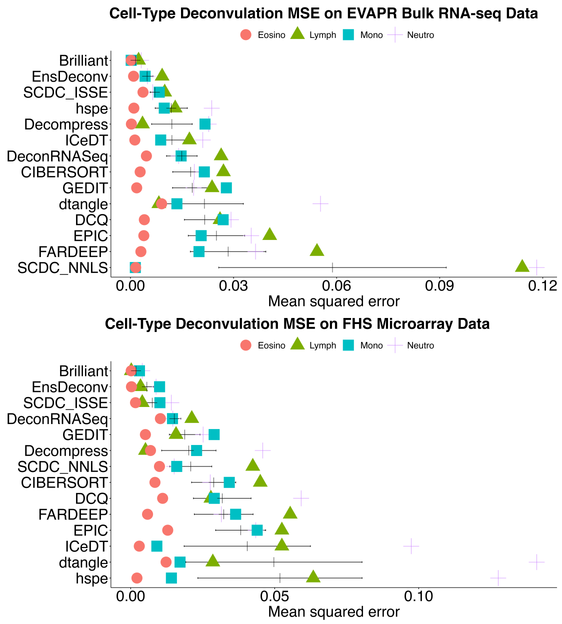

Fig. 4. Comparison of cell-type fraction prediction mean squared error (MSE) between Brilliant and different deconvolution methods in the EVAPR and FHS studies. Neutro is neutrophil; Mono is monocyte; Lymph is lymphocyte; and Eosino is eosinophil. Dot denotes the MSEs between the predicted fraction and truth for one specific cell type. The black vertical bar shows the mean of cell-type specific MSEs, and the horizontal line presents mean ± standard error of the mean.

$b_t hr$ is set to be the one achieves the smallest Mean Squared prediction Error (MSE) on the validation set.

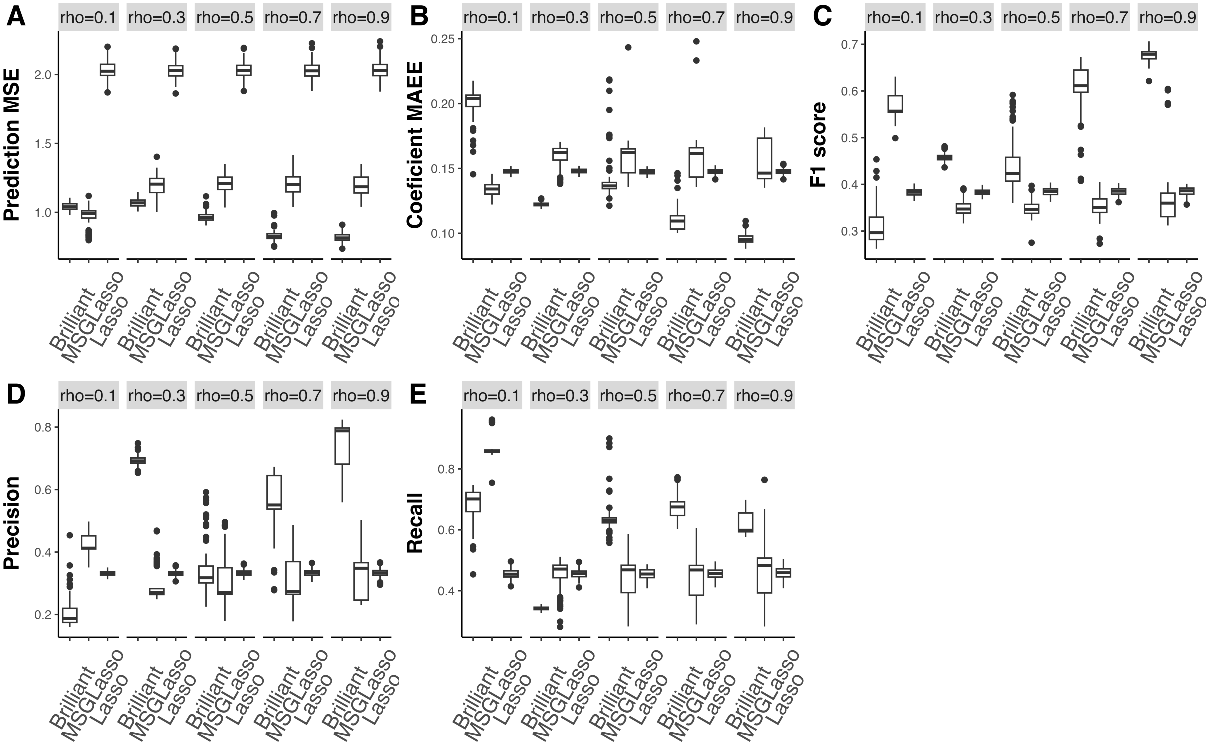

Fig. 5. Simulation results were obtained for $p = 500$ with 10 $\mathbf{X}$ groups and $q = 100$ in 5 $\mathcal{Y}$ groups. The covariance structure in each $\mathbf{X}$ group follows compound symmetry (CS) with a correlation coefficient of $\rho = 0.5$. In each $\mathcal{Y}$ group, the covariance structure also follows CS, with correlation coefficients ρ ranging from 0.1 to 0.9. The training sample size is 200, and the test sample size is 100. **A**. Mean squared prediction error (MSE), where a lower value indicates better prediction performance. **B**. Mean absolute estimation error of the coefficient matrix (MAEE), with a lower value suggesting better estimation. **C**. F1 score for feature selection. **D**. Precision for feature selection. **E**. Recall for feature selection. For **C**-**E**, a higher value indicates better feature selection performance.

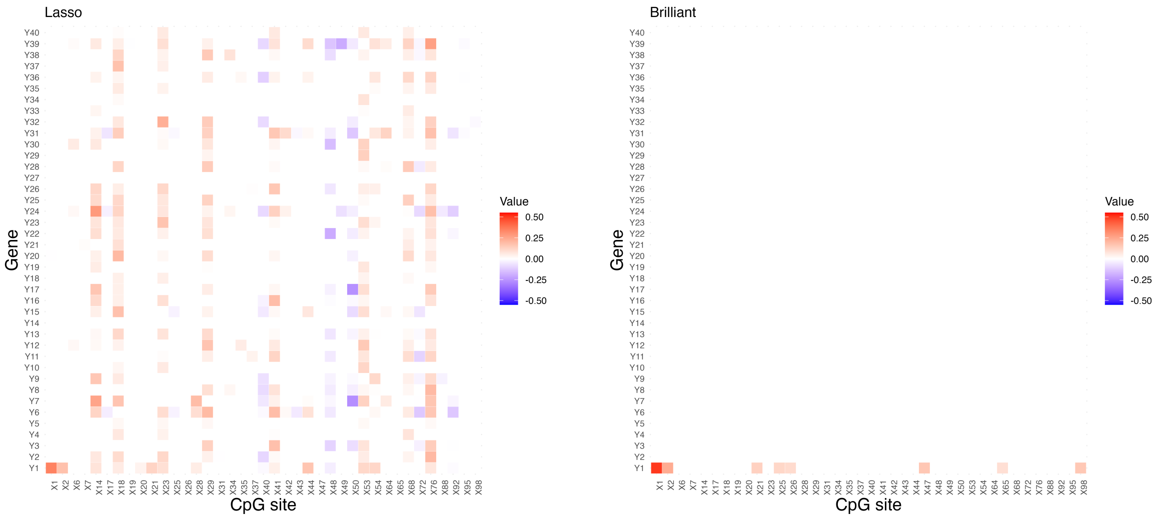

Fig. 6. Heatmaps of estimated coefficient to demonstrate Lasso select more false positives when the multivariate outcomes are correlated. Note: Predictors without any nonzero estimated coefficients by both Lasso and Brilliant are omitted in the graph.

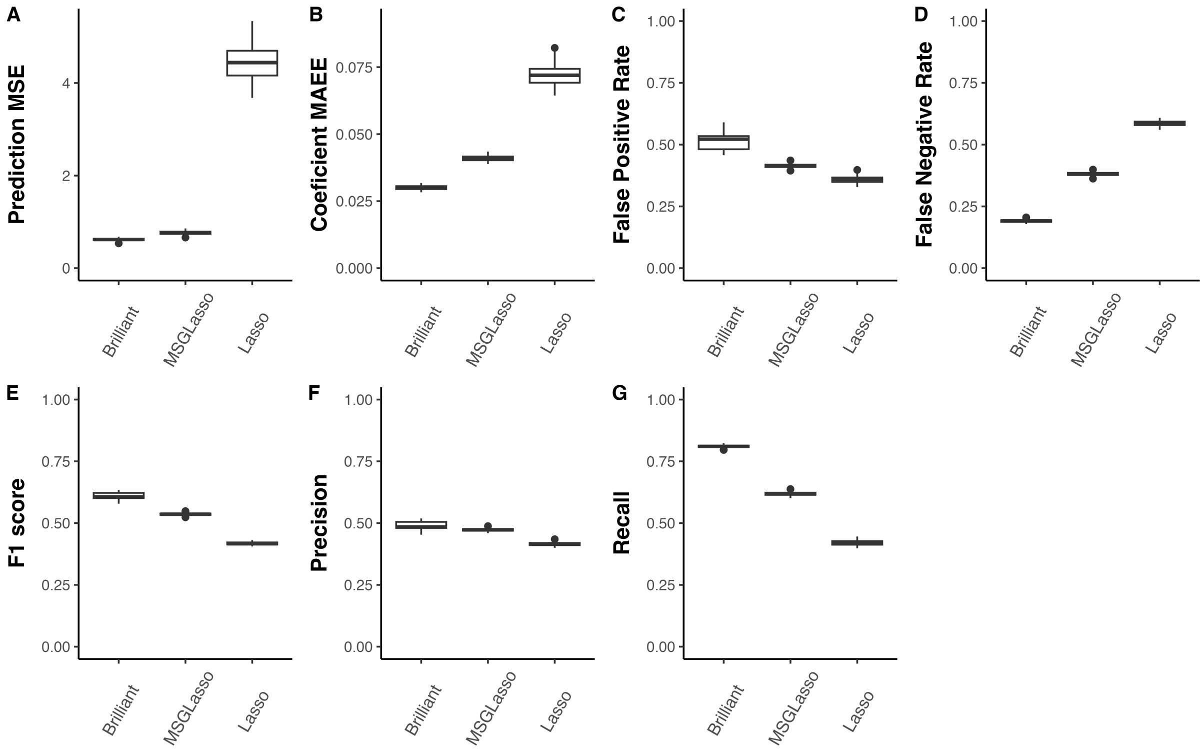

Fig. 7. Simulation results demonstrate that Brilliant has a superior ability to detect weak association signals.

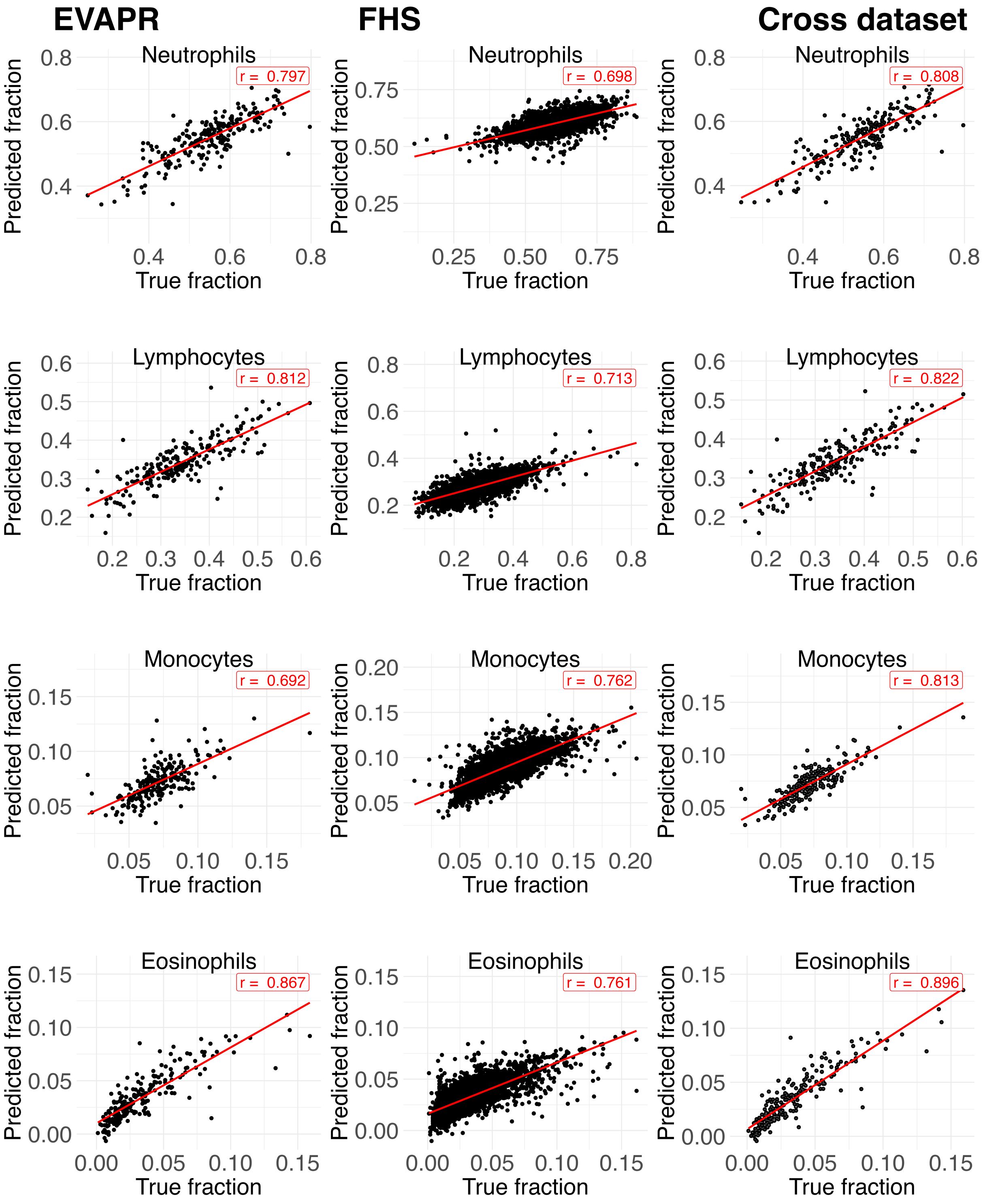

Fig. 8. Scatter plots of predicted v.s. referenced cell type fractions. The left panel plots are from CV using the EVAPR data only. The middle panel are from CV using the FHS data only. The right panel are from using EVAPR data as the test set while using the FHS data as the training set.

Table 2. Signature genes available in EVAPR and FHS dataset.

Dataset	Total number of genes	Neutrophil	Lymphocyte	Monocyte	Eosinophil
EVAPR	200	64	53	40	39
FHS	196	61	40	40	55
Overlap	188	59	51	37	37

* The values denote the number of signature genes.

* The overlapping genes in the two datasets are used to build the final model using the FHS data, and predict the cell type fractions for the EVAPR data.

Table 3. Top performers of cell type deconvolution for EVAPR dataset.

Method	Neutrophil	Lymphocyte	Monocyte	Eosinophil	Mean
Brilliant	0.80	0.81	0.69	0.87	0.79
Brlliant (Cross dataset)	0.77	0.79	0.71	0.89	0.79
EnsDeconv	0.80	0.81	0.69	0.82	0.78

* The values under each cell type are cell type specific Spearman's correlation. Mean denotes the average of the four cell-type specific Spearman's correlation.

* Brilliant (Cross dataset) shows the performance of the final model (built using the FHS data) on predicting the cell type fractions for the EVAPR data.

Table 4. Extra EVAPR data analysis results: Spearman's correlations between the predicted and measured cell-type proportions.

Method	Neutrophil	Lymphocyte	Monocyte	Eosinophil
Brilliant	0.797	0.811	0.691	0.866
MSGLasso	0.817	0.832	0.566	0.847
Lasso	0.819	0.834	0.676	0.860

Table 5. Extra FHS data analysis results: Spearman's correlations between the predicted and measured cell-type proportions.

Method	Neutrophil	Lymphocyte	Monocyte	Eosinophil
Brilliant	0.698	0.764	0.713	0.761
MSGLasso	0.745	0.768	0.772	0.776
Lasso	0.746	0.774	0.773	0.783
